# Metabolic Reprogramming and Renal Fibrosis

**DOI:** 10.3389/fmed.2021.746920

**Published:** 2021-11-08

**Authors:** Xiaoyu Zhu, Lili Jiang, Mengtuan Long, Xuejiao Wei, Yue Hou, Yujun Du

**Affiliations:** ^1^Department of Nephrology, The First Hospital of Jilin University, Changchun, China; ^2^Physical Examination Center, The First Hospital of Jilin University, Changchun, China

**Keywords:** renal fibrosis, chronic kidney disease, metabolic reprogramming, fatty acid, aerobic glycolysis

## Abstract

There are several causes of chronic kidney disease, but all of these patients have renal fibrosis. Although many studies have examined the pathogenesis of renal fibrosis, there are still no effective treatments. A healthy and balanced metabolism is necessary for normal cell growth, proliferation, and function, but metabolic abnormalities can lead to pathological changes. Normal energy metabolism is particularly important for maintaining the structure and function of the kidneys because they consume large amounts of energy. We describe the metabolic reprogramming that occurs during renal fibrosis, which includes changes in fatty acid metabolism and glucose metabolism, and the relationship of these changes with renal fibrosis. We also describe the potential role of novel drugs that disrupt this metabolic reprogramming and the development of fibrosis, and current and future challenges in the treatment of fibrosis.

## Introduction

Chronic kidney disease (CKD) has a worldwide prevalence of 10–15% (1), and its incidence has increased during recent years. The Global Burden of Diseases, Injuries, and Risk Factors Study (GBD) classified CKD among the 12 leading causes of death of 133 analyzed diseases (2). Following the development of CKD, some patients eventually progress to end-stage renal disease (ESRD) (3), and these patients can only survive by receipt of dialysis or kidney transplantation. CKD patients also face high medical costs, thus making CKD a huge burden to society and the families of affected patients (4). There are still no effective drugs that can effectively delay or reverse the progression of CKD, so new treatments are urgently needed. The most common causes of CKD are diabetes and high blood pressure, but all patients experience the pathological changes of renal fibrosis (5). Thus, it may be possible to develop novel antifibrotic interventions or drugs that delay or reverse CKD. However, renal fibrosis is a complex process involving a variety of cells, cytokines, and signaling pathways (6), and there are currently no effective methods to stop its progression. This highlights the need to develop a deeper understanding of the mechanisms of renal fibrosis and to translate that knowledge into new therapies.

CKD is the common outcome of renal fibrosis, but renal fibrosis may occur by several known pathways or may be idiopathic. For example, renal fibrosis may be secondary to glomerular injury and atrophy of the tubules that are fed by the injured glomerulus. This is associated with changes in hemodynamics (flow into the peritubular capillaries from the glomerular capillaries) and metabolism (impaired urine formation). This leads to glomerular vascular endothelial damage, glomerulosclerosis, and impaired glomerular secretion of transforming growth factor (TGF-β1), thus inducing atrophy of renal tubules and tubulointerstitial fibrosis (7). Atrophied renal tubules disrupt glomerular function, resulting in glomerular dysfunction and reduced urine filtration.

Renal fibrosis may also be caused by obstruction of the urinary outlet at any level. In this case hemodynamic changes are less important, but there is impaired urine flow and increased intraluminal pressure that induces tubular atrophy and fibrosis. In renal disease, the capillaries around the renal tubules are sparse, which leads to the reduction of blood, oxygen, and nutrition supplied to the renal tubules, and this contributes to renal fibrosis (8). Urinary tract obstruction also leads to kidney enlargement, urine accumulation in the renal pelvis or calyces, hydronephrosis, and then to progressive damage of renal structure and chronic renal insufficiency. At present, the unilateral ureteral obstruction (UUO) model is the most widely used model to study the mechanism of renal tubulointerstitial fibrosis.

Renal fibrosis may also be caused by drugs or other toxic substances that alter renal filtration and excretion and lead to renal injury. For example, aristolochic acid can lead to renal tubular atrophy and fibrosis, and eventually to end-stage renal disease (9). Therefore, renal fibrosis can also be considered as tubulointerstitial fibrosis. Although the etiology of renal fibrosis is complex and diverse, it is similar to other fibrotic diseases, such as liver fibrosis and pulmonary fibrosis (10).

The main pathogenic features of renal fibrosis are also increased myofibroblast activity (11), increased cytokine production and signaling (12, 13), inflammation (14), and hypoxia (15, 16). The rapid development of methods used to study cancer cells, metabolism, and metabolomics has led to increased focus on the reprogramming of energy metabolism in many diseases (17–19). Normal energy metabolism is the basis for maintaining the structure and function of tissues, and abnormalities can directly lead to pathological changes (20).

The main functions of the kidneys are the formation and excretion of urine, and these physiological processes regulate the stability of the body's water, electrolytes, and acid-base balance. At rest, the abundance of mitochondria and amount of oxygen consumption by the kidneys are second only to those in the heart (21). However, there is heterogeneity in the oxygenation of different regions of the kidneys and in the responses of different regions of the kidneys to hypoxia (22, 23). During normal function, the kidneys overall consume large amounts of energy (1, 24). Normal and regulated energy metabolism are prerequisites for maintaining the structure and function of the kidneys (25), and changes in energy metabolism may contribute to the pathogenesis of renal fibrosis (26).

The kidneys are composed of diverse cells that have different functions. Recent research on renal metabolic reprogramming has mainly focused on renal interstitial models and renal proximal tubules (27–29). At present, there is little known about metabolic reprogramming in the renal medulla, although this innermost region of the kidney requires significant energy to support excretion and transport functions. The proximal tubule cells account for 90% of the renal cortex and are the main adenosine triphosphate (ATP) consuming cells in the kidneys (30). Under normal physiological conditions, the renal tubular epithelial cells contain abundant mitochondria (31). These cells mainly rely on fatty acids (FAs) as fuel, and they generate energy by mitochondrial oxidative phosphorylation (OXPHOS) (32). When the kidneys are damaged, the renal tubules have altered energy metabolism (33), in that they generate energy by aerobic glycolysis (34). Many recent studies showed that metabolic reprogramming occurs in cancer cells, and also plays an important role in the onset and progression of fibrosis (35). Previous studies also showed that changes in energy metabolism occur during renal fibrosis (28, 36), and that changes in renal energy metabolism can lead to renal fibrosis (3). Alterations in energy metabolism and renal fibrosis thus promote each other, as a positive feedback cycle. Because normal energy metabolism is necessary for cell growth, proliferation, and other cellular activities (37, 38), identification of the metabolic alterations in fibrosis may help in the development of new and effective treatments (39).

## Fatty Acid Oxidation and Renal Fibrosis

Lipid metabolism is closely related to kidney disease, and CKD patients often have abnormal lipid metabolism (40). Dyslipidemia may be a consequence or a cause of kidney disease (41, 42). Normal renal tubular epithelial cells consume large amounts of energy (43). The catabolism of one molecule of a FA produces much more ATP than the catabolism of one molecule of glucose (44), and healthy renal tubular epithelial cells mainly rely on FA oxidation (FAO) to produce energy (45). FA metabolism consists of anabolism and catabolism (37), and the synthesis of FAs typically starts from acetyl CoA, and is completed by FA synthase and acetyl CoA hydroxylase (46). Unused FAs are mainly stored as triglycerides. As the amount of triglycerides increases, excessive lipid droplets can form inside cells, causing cell lipid toxicity, a response that may contribute to the development of fibrosis (47). Proteinuria is also associated with renal fibrosis, and Kees-Folts et al. (48) proposed that this relationship was attributable to FA accumulation because tubular catabolism of albumin was related to the release of pro-inflammatory lipids. Most FAs are catabolized in the mitochondria, and peroxidase plays a central role (49). Long chain FAs enter the renal tubular cell cytoplasm *via* a FA transport enzyme (CD36) and a FA binding protein (FABP) (43). In the cytoplasm, fatty acyl CoA synthase activates FAs by forming fatty acyl CoA (50). Cells then transport acyl CoA into the mitochondria *via* carnitine acyltransferase I and II, and FAO produces acetyl CoA (51), a fuel for the tricarboxylic acid (TCA) cycle and OXPHOS (52). Lipid synthesis and catabolism are highly regulated in healthy renal tubular epithelial cells (53). However, an imbalance between FA intake and utilization can lead to lipid accumulation and kidney damage (54).

FAs are essential for cell signaling, energy production, and regulation of inflammation (55). Since the “lipid nephrotoxicity hypothesis” was first proposed in 1982 (56), there has been increasing evidence that supports the close relationship between altered lipid metabolism and kidney disease (57). In particular, studies of patients and animal models of renal interstitial fibrosis reported that the key enzymes regulating FAO had reduced function, and that this led to increased intracellular lipid deposition, thus confirming that reduced FAO activity is a strong determinant of renal fibrosis (58–60). For example, mice fed a high-fat diet (HFD) accumulated lipids in the kidneys, and this led to structural damage of tubular epithelial cells, inflammation, and fibrosis (61, 62). Inhibition of FAO leads to lipid deposition in the renal epithelial cells, which then transform into stromal cells, leading to inflammation and interstitial fibrosis (63). In most fibrous tissues, transforming growth factor β1 (TGF-β1) downregulates peroxisome proliferator-activated receptors (PPARs), which function in FAO (64). The increase of FA synthesis can increase TGF-β1 and reduce extracellular matrix (ECM) degradation.

In agreement, increasing FAO in mice protected them from tubulointerstitial fibrosis (65). Kang et al. (66) suggested that the drug etomoxir (an FAO inhibitor) had pro-fibrotic effects. Treatment of mice that had renal fibrosis with fenofibrate (a PPAR-α agonist) significantly up-regulated the expression of genes that mediate FAO [carnitine palmitoyl-transferase 1 (*CPT1*), carnitine palmitoyl-transferase 2 (*CPT2*), acyl-CoA oxidase 1 (*ACOX1*), and acyl-CoA oxidase 2 (*ACOX2*)], reduced fibrosis, and improved renal function. Clofibrate (67), a drug that enhances FAO, may also have a therapeutic effect in patients with renal fibrosis. In general, fibrates increase lipolysis and reduce triglyceride accumulation by inducing the expression of specific FA transporters and increasing the uptake of free FAs by the liver (68). However, due to their adverse effects on renal function, PPAR-α agonists are not commonly administered to patients with kidney disease.

## Aerobic Glycolysis and Renal Fibrosis

Previous studies found that the energy metabolism of renal tubular epithelial cells changes during the progression of renal fibrosis. Healthy cells generally use FAO and OXPHOS, but diseased cells have greatly increased glycolysis (36). Similar to the “Warburg effect” in tumor cells (see below) (69). Glucose is normally the main source of energy for most cells (70). Glucose metabolism begins with cellular uptake, and proteins in the glucose transporter family (GLUT1, GLUT2, GLUT3, and GLUT4) are responsible for transferring glucose into the cytoplasm through the plasma membrane (71). Then, glycolysis converts glucose into pyruvate *via* hexokinase (HK2), phosphofructokinase (PFK1), glyceraldehyde 3-phosphate dehydrogenase (GAPD), phosphoglycerate kinase (PGK), and pyruvate kinase M2 (PKM2) (72). HK2, PFK1, and PKM2 are the key regulatory enzymes in glycolysis (73–75). Under normoxia, most eukaryotic cells convert pyruvate into acetyl CoA *via* pyruvate dehydrogenase (PDH); the acetyl COA then enters the tricarboxylic acid cycle (TCA) and OXPHOS eventually generates ATP. Under hypoxia, cytoplasmic lactate dehydrogenase A (LDHA) converts pyruvate into lactic acid (76, 77).

About 100 years ago, Warburg et al. observed that certain cancer cells rely on glycolysis for rapid energy production, even in normoxic environments. Subsequent studies identified the “Warburg effect” in other cancers (78–80). Interestingly, recent studies also reported that the Warburg effect also occurs in non-tumor diseases, such as inflamed tissues (75, 81), pulmonary hypertension (82, 83), polycystic kidney disease (84, 85), and fibrosis of various tissues and organs (18, 26, 86, 87). Importantly, the metabolites produced by aerobic glycolysis alter the regulation of key cell functions, including cell proliferation, production of extracellular matrix, autophagy, and apoptosis (88–90). Thus, the Warburg effect has an impact far beyond energy generation.

There has been a rapid increase in research on the role of energy metabolism in fibrosis, and it is now established that altered energy metabolism plays an important role in the process of renal fibrosis. In particular, new evidence from patients with CKD and animal models of fibrosis (29, 91–93) identified a metabolic shift from mitochondrial OXPHOS to aerobic glycolysis during kidney fibrosis. At the molecular level, renal tissues and fibroblasts have increased expression of glycolytic enzymes (HK2, PKM2, LDHA) and lactic acid production during fibrosis (20, 27, 93–95). Wei et al. (34) studied mice with unilateral ureteral obstruction (UUO) and demonstrated that two agents that disrupted glycolysis, shikonin (an inhibitor of PKM2) and dichloroacetate [DCA; an inhibitor of pyruvate dehydrogenase kinase-1 (PDK1)], significantly inhibited renal fibrosis, renal tubular apoptosis, and renal inflammation. They also showed that these two agents had different effects on renal fibroblasts and renal tubular cells. Therefore, the type and location of cells altered by an anti-glycolytic agent should be considered when choosing a treatment that inhibits glycolysis and prevents renal fibrosis.

Ding et al. (28) also studied mice with UUO and reported that the metabolic conversion from OXPHOS to aerobic glycolysis was the main feature of fibroblast activation, and that inhibition of aerobic glycolysis by shikonin and 2-deoxyglucose (2-DG) alleviated this effect and the TGFβ-1-mediated activation of myofibroblasts. Liu et al. (96) showed that a PKM2 agonist (TEEP-46) inhibited renal fibrosis by blocking the epithelial-mesenchymal transition (EMT) and the abnormal activation of glycolysis. All these findings confirm that alterations in cell metabolism play a central role in renal fibrosis, and suggest that drugs which stimulate FAO or inhibit glycolysis may be effective treatments.

## Metabolic Reprogramming and Myofibroblast Activation

The overproduction of ECM by myofibroblasts and the inhibition of its degradation are essential features of renal fibrosis (97). Myofibroblasts are mainly activated or undergo phenotypic transformation (such as the EMT) by transformation of fibroblasts, renal tubular epithelial cells, endothelial cells, mesenchymal stem cells, and some other cells (98, 99). Recent studies showed that the activation and proliferation of fibroblasts during renal interstitial fibrosis is similar to the metabolic reprogramming in cancer cells (100). Metabolic reprogramming is generally recognized as a key process for the activation of fibrotic cells in different organs (101). The balance of FAO and glycolysis affects the production and degradation of the ECM. In particular, FAO can directly promote degradation of the ECM (39), and Zhao et al. (102) found that enhancing FAO reduced the accumulation of ECM and skin fibrosis. Glycolysis also has a key role in determining the fibrogenic phenotype of fibroblasts (103). Increased glycolysis is necessary for the formation and production of myofibroblasts (87), and provides the metabolites needed for ECM biosynthesis (88). Bernard et al. (104) were the first to demonstrate that the differentiation of fibroblasts into myofibroblasts was accompanied by significant metabolic reprogramming, and that this reprogramming was a sign of myofibroblast differentiation that was essential for the contractile function of these cells. Xie et al. (105) proposed that enhanced glycolysis consistently occurred early during the differentiation of myofibroblasts, and was mainly dependent on the increased expression of the key glycolytic enzyme 6-phosphofructo-2-kinase/fructose 2,6-bisphosphatase 3 (PFKFB3). They also showed that inhibiting PFKFB3 inhibited glycolytic flux, myofibroblast differentiation, and pulmonary fibrosis.

## Metabolic Reprogramming and Renal Fibrosis-Related Cytokines and Signaling Pathways

There are alterations in many cytokines and signaling pathways during the pathogenesis of renal fibrosis (106), and TGF-β1 is a main driver of this pathogenic process (107). TGF-β1 promotes the proliferation and activation of fibroblasts into myofibroblasts, and also induces the EMT, thus increasing the abundance of myofibroblasts (108, 109). Yadav et al. (110) found that TGF-β1 inhibited the key transcription factor peroxisome proliferator-activated receptor-γ coactivator 1α (PGC1-α; an important regulator of FAO) and increased downstream lipid deposition in a Smad3-dependent manner. Kang et al. (66) found that TGF-β1 reduced the expression of enzymes related to FAO in a SMAD3 and PPAR γ coactivator-1α (PPARGC1A) -dependent manner, and that fenobibrate (a PPAR-α agonist) reversed the downregulation of FAO, CPT1, and ACOXS. A study of diabetic mice reported that increased triglyceride synthesis was related to an increased level of TGF-β1 (111). In addition to affecting lipid metabolism, TGF-β1 is also a strong activator of aerobic glycolysis. In particular, TGF-β1 can directly induce glycolytic enzymes, such as HK2 and PFK, and enhance glycolysis by stabilizing hypoxia inducible factor 1 (HIF-1) (112, 113). Yin et al. (20) studied rat fibroblasts and reported that TGF-β1 induced the metabolic conversion from OXPHOS to aerobic glycolysis; they also reported a decreased pH in TGF-β1-treated culture medium, with increases in lactate concentration and glucose consumption over time.

Srivastava et al. (114) studied renal tubular epithelial cells and found that TGF-β1 increased the expression of glycolytic enzymes (HK2, PKM2, PDK4, and GLUT1) and inhibited the levels of FAO-related enzymes (PGC1α and CPT1A). Hua et al. (115) showed that TGF-β1 led to enhanced glycolysis during the EMT and that glycolysis also functioned in regulating TGF-β1 production. In particular, there is evidence that glycolysis and lactic acid promote the differentiation of myofibroblasts and enhances the secretion of TGF-β1 (105, 116). TGF-β1 stimulates the production of lactic acid, and lactic acid accumulation decreases the pH in the microenvironment, which further enhances the activity of TGF-β1 and increases the differentiation of cells into myofibroblasts (89, 117). This positive feedback loop indicates the potential role oβf TGF-β1 as an important metabolic regulator. Direct targeting of TGF-β is one possible strategy for alleviating fibrosis, and drugs that inhibit TGF-β synthesis, activation, or downstream signaling are available. For example, nintedanib and pirfenidone can be used for treatment of idiopathic pulmonary fibrosis. However, the toxic effects from direct antagonism of TGF-β can be significant, and many patients cannot tolerate these drugs. Therefore, from the perspective of metabolism, inhibition of the positive feedback loop between TGF-β and energy metabolism may be a safer and more effective strategy.

The Wnt/β-catenin signaling pathway is an important evolutionarily conserved developmental pathway that regulates cell development, growth, metabolism, and tissue homeostasis and damage repair processes (118), Although Wnt/β-catenin signaling is relatively silent in normal adult kidneys, kidney diseases in humans and animal models are associated with activation of this pathway (119, 120). During fibrosis, an interaction between TGF-β1 and the Wnt/β-catenin pathway reprograms energy metabolism (121). The Wnt/β-catenin pathway is a critical regulator of glycolytic energy metabolism in fibroblasts (122), and its upregulation enhances the levels of HK2, PKM2, PDK-1, LDHA, and monocarboxylate lactate transporters (MCTs), leading to increased lactate production and secretion (123, 124). The activation of the PI3K/AKT signaling pathway can also promote aerobic glycolysis (125), and the Wnt/β-catenin pathway stimulates the PI3K/Akt pathway to further promote glycolysis (126).

AMP-activated protein kinase (AMPK), a member of the serine/threonine kinase family, is an important sensor of cellular energy (127) that functions in a variety of organs (128). This enzyme plays an important role in several metabolic pathways, including glycolysis and the oxidation and synthesis of FAs (129). Previous studies showed that AMPK reduced renal fibrosis by antagonizing TGF-β1/Smad3 signaling and inhibiting the EMT (130, 131). AMPK is activated when ATP is consumed or when the intracellular AMP/ATP ratio is high (132). Once activated, AMPK acts on downstream PPAR and PGC1-α, promotes mitochondrial FA uptake and oxidation, reduces FA synthesis (110, 133), and reprograms the metabolism from OXPHOS to aerobic glycolysis (69). In addition, AMPK can increase GLUT4 translocation and glucose uptake (134). There is evidence that AMPK regulates glycolysis by phosphorylating and activating phosphofructokinase 2 (135). The rapamycin target mTOR is one of the downstream targets of AMPK, and it functions as a receptor for ATP, thereby balancing nutrient availability and cell growth (136, 137). mTOR also regulates the expression of TGF-β1, is closely related to the EMT, and plays an important role in renal fibrosis. Tian et al. (138) found that inhibition of AMPK activated mTOR and increased the levels of TGF-β1 and pro-fibrotic proteins.

Autophagy, a normal catabolic process in which lysosomes degrade most of the cytoplasmic contents, is essential for maintaining renal homeostasis, structure, and function (139). Yamamoto et al. (140) studied renal proximal tubular cells and found that downregulation of autophagy was associated with lipid toxicity. AMPK is a key regulator of autophagy, and AMPK-mediated activation of autophagy provides protection from renal injury (141). Thus AMPK signaling may be a novel therapeutic target for the treatment of fibrotic nephropathy.

## Metabolic Reprogramming and Inflammatory Cells in Renal Fibrosis

Fibrosis is usually associated with a strong inflammatory response and the infiltration of immune cells. Previous studies showed that damaged renal tubular epithelial cells recruited inflammatory cells to the renal interstitial compartment, and these inflammatory cells then produced numerous pro-inflammatory and pro-fibrotic cytokines (142). Macrophages play an important role in renal injury, and in the repair, maintenance, and stability of the internal renal environment (143). The two main subtypes of macrophages are M1 (classically activated macrophages) and M2 (alternatively activated macrophages), and each subtype can transform into the other (144). Metabolism plays a central role in regulating the physiological effects of the M1 and M2 phenotypes. M1 macrophages mainly rely on glycolysis, and M2 macrophages mainly rely on OXPHOS (145). As CKD becomes increasingly aggravated, M2 macrophages transform into M1 macrophages, and this accompanies the metabolic reprogramming from OXPHOS to glycolysis (1).

T cell infiltration occurs in most chronic diseases. For example, studies of humans and experimental animals with nephropathy indicated that tubular interstitial fibrosis was related to the interstitial infiltration of T cells and macrophages (146). T cells are activated during kidney injury and may directly promote the production and activation of myofibroblasts, induce monocyte recruitment, increase the inflammatory response, and induce the pro-fibrotic phenotype of macrophages (147). Metabolic reprogramming is closely related to the growth, development, activation, differentiation, and function of T cells (148). A study of the mitochondria of monocytes and macrophages showed that the process of T cell activation and cytokine production was accompanied by a metabolic conversion from OXPHOS to aerobic glycolysis (149).

## Metabolic Reprogramming and Hypoxia in Renal Fibrosis

The complex structure of the kidney is related to heterogeneity in the oxygenation of different regions (22). Under normal physiological conditions, pO_2_ sharply declines at the cortico-medullary junction, and is as low as 20 mmHg within the renal medulla (23). The responses of different regions of the kidney to hypoxia also differ. The medullary thick ascending limbs (mTALs) play a central role in maintaining systemic acid-base and electrolyte homeostasis (150). The mTALs and straight segment of the proximal tubule have a strong demand for extramedullary oxygen transport from the renal tubules, which are very vulnerable to hypoxia (22).

Hypoxia is a major feature of the microenvironment of fibrotic tissues, and hypoxia-inducible factor 1 (HIF-1) is a heterodimeric nuclear transcription factor that plays an important role in the responses of cells to hypoxia (151). HIF-1 promotes cell adaptability to hypoxia and functions in tissue protection under conditions of hypoxia. HIF-1 is expressed in tubular cells, and its accumulation varies among nephron segments. Compared with the collecting ducts, mTALs have limited ability to produce HIF-1, which may explain their greater susceptibility to injury (152). HIF-1 consists of an oxygen-sensitive subunit (HIF-1α) and a constituent active subunit (HIF-1β) (153). In normoxia, HIF-1α is hydroxylated by proline hydroxylase and subsequently degraded by proteasomes; in hypoxia, there is inactivation of proline hydroxylase, leading to the stabilization and translocation of HIF-1α into the nucleus, where it fuses with HIF-1β to form an active HIF-1 transcription factor (154). In addition to promoting cell adaptation to hypoxia, HIF-1 can increase the expression of α-SMA, and when the level of HIF-1 exceeds a certain level, this leads to activation of myofibroblasts (105).

**Figure 1:**
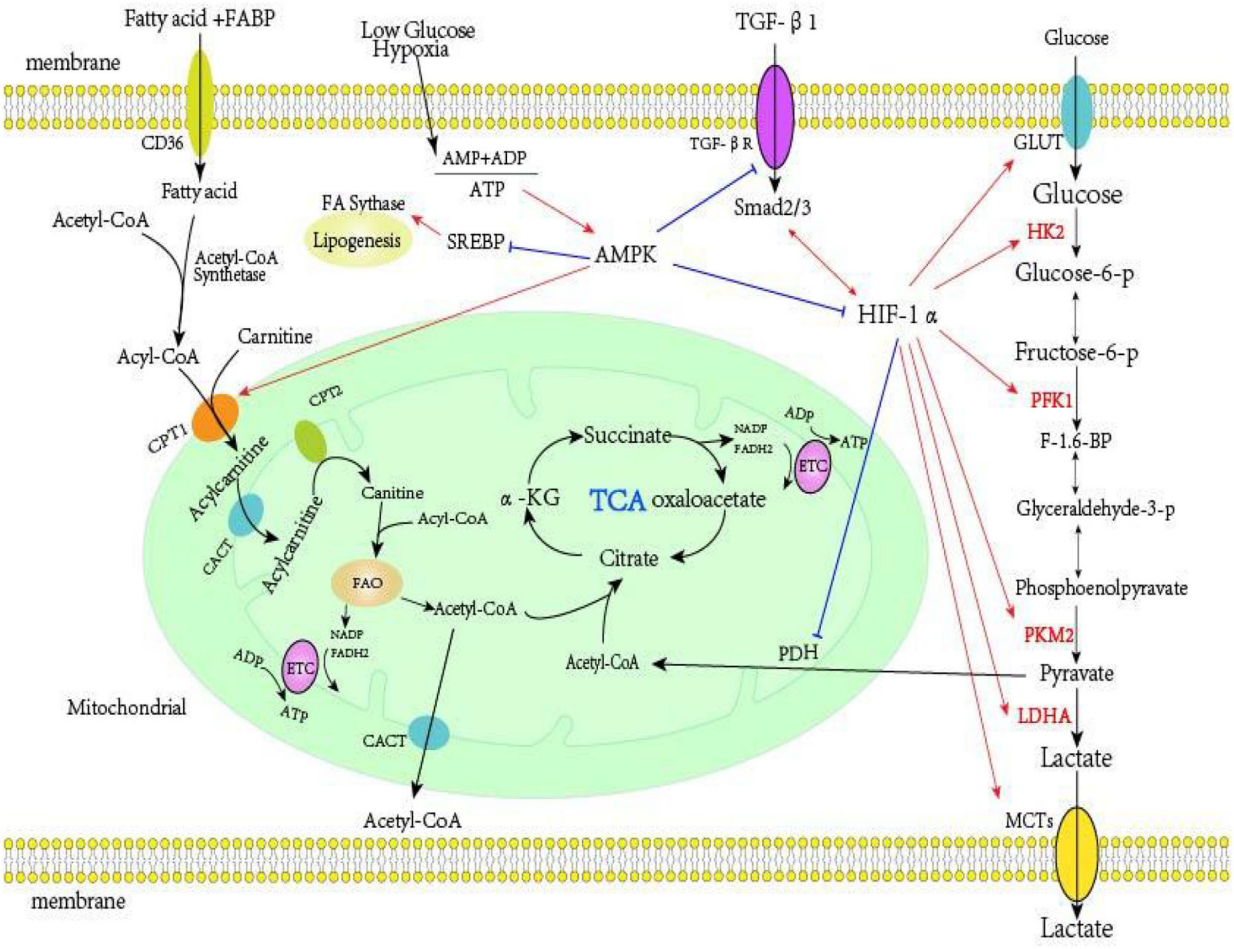
Metabolism in renal proximal tubule cells. Far-left: FAs are the preferred energy substrates for the kidney. FAs bound to FABP are transported into the proximal tubule cells via platelet glycoprotein 4 (also known as CD36). In the cytosol, FAs are converted from acetyl-CoA to acyl-CoA by acetyl-CoA synthetase, then transferred to the mitochondrial matrix via the carnitine shuttle (CPT-1, CACT, and CPT-2). The acyl-CoA undergo β-oxidation to generate acetyl-CoA, which fuels the TCA cycle and also generates NADH and FADH2, which function serve as electron donors to the ETC on the IMM for ATP production. Far-right: Glucose enters the cell via GLUT transporters. In the intracellular environment, glycolysis converts glucose into pyruvate, a process controlled by several rate limiting enzymes (HK2, PFK1, and PKM2). Under hypoxia, cells usually convert pyruvate into lactic acid via lactate dehydrogenase. Under normoxia, pyruvate enters the TCA. Middle-right: HIF-1α is the key regulator of glycolysis, and it increases the expression of HK2, PFK1, PKM2, LDHA, and PDK1, and reduces the expression of PDH. TGF-β1 is a master regulator of renal fibrosis, and TGF-β1, HIF-1α, lactic acid, and LDHA can form a positive feedback loop that increases aerobic glycolysis. Middle: AMPK, an intracellular energy sensor that maintains the energy balance within the cell, is activated when the AMP+ADP/ATP ratio increases. AMPK can inhibit the activity of TGF-β1 and HIF-1α, and also inhibit fatty acid synthesis. AMPK induces fatty acid import into the mitochondria by inactivating malonyl-CoA production, thereby alleviating inhibition of CPT1, the rate-limiting enzyme for mitochondrial fatty acid oxidation. FA, fatty acid; FABP, fatty acid-binding protein; CPT-1, carnitine palmitoyltransferase I; CACT, carnitine-acylcarnitine; CPT-2, carnitine palmitoyltransferase II; TCA, tricarboxylic acid cycle; NADH, nicotinamide adenine dinucleotide; FADH2, flavin adenine dinucleotide; ETC, electron transport chain; IMM, mitochondrial inner membrane; ATP, adenosine triphosphate; GLUT, glucose transporter; HK2, hexokinase; PFK1, phosphofructokinase; PKM2, pyruvate kinase M2; HIF-1α, hypoxia-inducible factor 1α; LDHA, lactate dehydrogenase A; PDK1, pyruvate dehydrogenase kinase-1; PDH, pyruvate dehydrogenase; TGF-β1, transforming growth factor-β1; AMPK, AMP-activated protein kinase.

HIF is also a central regulator of glycolytic energy metabolism. At the molecular level, HIF-1 promotes overexpression of the glucose transporter (GLUT), thereby increasing glucose absorption; it also activates glycolytic enzymes (HK2, PKM2, and LDHA), and induces PDK1 to phosphorylate PDH, thereby preventing entry of pyruvate into the TCA cycle and increasing the production of lactic acid (155, 156). Increased glycolysis disrupts the TCA cycle, resulting in an increased level of succinic acid (37). Succinic acid can stabilize HIF-1α, increase TGF-β1-induced HIF-1α expression, and promote fibrosis independently of hypoxia (157). Xu et al. (158) found that inhibition of HIF-1α significantly reversed the increased aerobic glycolysis in cancer cells. Other research found that an increase of HIF-1α inhibited the expression of key enzymes in FAO (PPARA and CPT1A), thereby downregulating FAO (159). Cai et al. (36) showed that increased expression of HIF-1α during the fibrosis of renal proximal tubules led to reprogrammed cell metabolism from FAO to glycolysis and lipid accumulation. Faubert et al. (160) studied tumor growth *in vivo* and reported that AMPK down-regulated the expression of HIF-1α and had an “anti-Warburg effect.” Another study of metabolic reprogramming during fibrosis (121) reported that HIF-1α, TGF-β, LDHA, and lactic acid formed a positive feedback loop, in which HIF-1α played a key role. In addition, previous research found that the use of a HIF stabilizer counteracted the change of energy metabolism that occurred during the early stages of diabetic nephropathy and that this had protective effect on pathophysiology of diabetic kidney disease (161). Other studies showed that HIF stabilizers ameliorated the progression of renal fibrosis (162, 163). Wu et al. (164) found that the HIF stabilizer FG-4592 retarded the progression of AKI to CKD by improving vascular regeneration and antioxidative capability. Therefore, targeting HIF-1α and metabolic reprogramming may be effective approaches for the treatment of fibrosis of the kidneys and other organs. Figure 1 summarizes the metabolism in renal proximal tubule cells.

At present, most studies of metabolic reprogramming during renal fibrosis have focused on renal interstitial fibrosis. Analogously, studies of cardiovascular diseases found that metabolic reprogramming was closely related to the function of vascular endothelial cells. Studies of aristolochic acid nephropathy may provide further insight because this condition is characterized renal tubular atrophy and fibrosis, a reduced ATP supply, and general disruption of cellular energy metabolism, leading to cell dysfunction and a cell energy crisis (9). Further study of this phenomenon may help to consolidate and expand our understanding of energy metabolism in the kidney.

## Potential Treatments

Chemical energy is necessary for cell survival, proliferation, and differentiation. This review described many direct or indirect effects of metabolic reprogramming during the pathogenesis of renal fibrosis. Interventions that target the metabolic changes described here have potential as treatments for fibrosis of the kidneys and other organs. Below, we provide a brief summary of recent studies of drugs designed to treat fibrosis by targeting cell metabolism (Table 1).

**Table 1:** Metabolic targets for inhibition and promotion of fibrosis.

**Target**	**Drug**	**Outcome**	**References**
AMPK	Metformin, AICAR, A-769662, 1,25(OH)2D3	Increase AMPK level and slow the progression of interstitial fibrosis.	Satrianoe et al. (165) Hinson et al. (166) Tian et al. (138)
	Compound C	AMPK inhibitor that blocks the beneficial effects of metformin.	Lee et al. (167)
PPAR-α	Fenofibrate	Agonist that induces the expression of FAO enzymes (CPT1/CPT2 and ACOX1/ACOX2) and restores FAO.	Tanaka et al. (168) Kang et al. (66)
	BAY PP1	Restores PPAR-α expression and significantly reduces tubulointerstitial fibrosis, proliferation of interstitial fibroblasts, and TGF-β1 expression.	Boor et al. (169)
	ATF6a	PPAR-α inhibitor that disrupts fatty acid metabolism in PTCs.	Jao et al. (170)
CPT1	C75	Increases CPT1 activity, blocks fatty acid synthase, reduces kidney fibrosis, and improves kidney function.	Kang et al. (66)
SGLT2	Dapagliflozin	Suppresses SGLT2 and reduces HIF-1α-mediated transition of FAO to glycolysis.	Cai et al. (36)
HK2	2-DG 3-BrPA	Inhibits HK2 and decreases glycolysis. Inhibits HK2 and decreases glycolysis.	Xie et al. (105) Ding et al. (28) Yu et al. (27)
PFKFB3	3PO	PFKFB3 inhibitor that blocks bleomycin- and TGF-β1-induced lung fibrosis in mice.	Xie et al. (105)
PKM2	Shikonin	Inhibits renal aerobic glycolysis by reducing phosphorylation of PKM2 and attenuates renal fibrosis.	Ding et al. (28)
PDK1	DCA	PDK1 inhibitor that abolishes TGF-β1-induced myofibroblast activation.	Ding et al. (28)

*AMPK, AMP-activated protein kinase; AICAR, 5-aminoimidazole-4-carboxamide; PPAR-α, peroxisome proliferator-activated receptor α; CPT, carnitine O-palmitoyltransferase; ACOX, acyl-coenzyme A oxidase; SGLT, sodium-glucose cotransporter; HK, hexokinase; 2-DG, 2-deoxyglucose; 3-BrPA, 3-bromopyruvate; PFKFB, 6-phosphofructo-2-kinase/fructose 2,6-bisphosphatase; 3PO, 3-(3-pyridinyl)-1-(4-pyridinyl)-2-propen-1-one; PKM, pyruvate kinase muscleisozyme; PDK, pyruvate dehydrogenase kinase; DCA, dichloroacetate; PTC, proximal tubule cells. The color (shaded) are inhibitors of up regulating factors related to fatty acid oxidation process, and color (un-shaded) are drugs that directly or indirectly promote fatty acid oxidation and inhibit glycolysis*.

Satriano et al. (165) studied an animal model of CKD established by subtotal nephrectomy and found that metformin or 5-aminoimidazol-4-formamide ribonucleotide (AICAR) led to increased AMPK activity, corrected the low metabolic efficiency of the kidneys, and ameliorated renal fibrosis and associated structural changes. Hinson et al. (166) studied an animal model of hypertrophic cardiomyopathy and demonstrated that an AMPK agonist (A769662) prevented fibrosis and cardiac hypertrophy by inhibiting TGF-β. Tian et al. (138) treated UUO rats with 1,25-(OH)2D3 and found that it increased the level of AMPK, reduced the level of mTOR, inhibited the over-activation of fibroblasts, and slowed the progression of renal interstitial fibrosis. Lee et al. (167) performed *in vitro* experiments and showed that metformin inhibited EMT by inducing heme oxygenase-1 and thioredoxin, which inhibited intracellular ROS production; they also showed that an AMPK inhibitor (compound C) blocked the protective effects of metformin on the EMT and fibrosis. The PPAR-α agonist fenofibrate disrupted the TGF-β1-mediated inhibition of FAO and induced the expression of enzymes that functioned in FAO (CPT1 and 2, and ACOX1 and 2) and normalized overall FAO (66, 168). During renal fibrosis in rats, treatment with BAY PP1 restored PPAR-α expression and significantly reduced tubulointerstitial fibrosis, the proliferation of interstitial fibroblasts, and TGF-β1 expression (169). Administration of a PPAR-α inhibitor (ATF6α) to mice altered FA metabolism in PTCs (170). Kang et al. (66) demonstrated that C75 increased CPT1 activity, blocked FA synthase, reduced kidney fibrosis, and improved kidney function. Other research found that caffeic acid upregulated PPAR genes, enhanced FAO, inhibited glycolysis, downregulated fibronectin and collagen-I, and reduced skin fibrosis in mice (102). Cai et al. (36) studied diabetic mice and showed that dapagliflozin suppressed SGLT2 and reversed the HIF-1α-mediated reprogramming from FAO to glycolysis. Notably, 2-DG can function as a competitive inhibitor of HK2 and thereby reduce TGF-β1-induced fibrosis, increase environmental pH, reduce lactic acid accumulation, and remarkably decrease glycolysis in myo-fibroblasts (28, 105). The glycolysis inhibitor 3-BrPA can protect against fibrosis in the liver. Yu et al. (27) studied the mouse UUO model and found that 3-BrPA effectively reduced the levels of enzymes related to aerobic glycolysis (HK2, PKM2, LDHA), and reduced the accumulation of ECM in a dose-dependent manner, thereby suppressing myo-fibroblast differentiation. A study of mice demonstrated that a PFKFB3 inhibitor (3PO) provided therapeutic benefit from bleomycin- and TGF-β1-induced lung fibrosis (105). Ding et al. reported that shikonin inhibited renal aerobic glycolysis by reducing the phosphorylation of PKM2 and also attenuated renal fibrosis (28), and that a PDK1 inhibitor (DCA) abolished TGF-β1-induced myofibroblast activation (28). In addition, some drugs entering clinical trials, such as agonists of PPAR (NCT02704403, NCT03459079, NCT03008070, lanifibranor, elanfibranor), drugs that stimulate insulin secretion (NCT70251431), oral insulin (ORMD0801), and others, have potential anti-fibrosis effects (39). Studies of the metabolic treatment of tumors found that a GLUT inhibitor (rapafusyn) (171), HK2 inhibitors [lonidamin (100), ketoconazole, and posaconazole (172)] and LDHA inhibitors [pidididione derivatives, miR-30a-5p, and miR-41 (173)] altered the metabolism and inhibited the growth of tumors. Although there is no evidence that these drugs reduce fibrosis of the kidneys and other organs, *in vitro* studies may provide insights into their use for treatment of fibrosis from the perspective of metabolic reprogramming. Of course, tissue and cell specificity of the responses must be considered.

Metabolism and the many physiological and pathological processes of the body are intertwined in a complex network. Preclinical and clinical studies are still needed to examine the effect of energy metabolism on fibrosis and the tolerance and effectiveness of drugs that alter metabolism. Our review indicates that interventions and drugs that focus on metabolic targets may make a significant contribution to the treatment of fibrotic diseases.

## Conclusion

Fibrosis is a common outcome of many chronic diseases, but there are no effective treatments for renal fibrosis. Regulation of cellular energy metabolism is essential for the survival, proliferation, differentiation, and function of cells and organisms, and the kidneys consume large amounts of cellular energy. There is now strong evidence that metabolic reprogramming occurs during and contributes to the process of kidney fibrosis. Studies of animal models and *in vitro* experiments have identified drugs that can ameliorate fibrosis. Although none of these drugs are currently used for the clinical treatment of fibrosis, the methods and drugs known to restore normal metabolic pathways *in vitro* and *in vivo* are likely prospects for the treatment of fibrosis of the kidneys and other organs in humans. However, because different organs and different cells within the same organ may be biased toward certain modes of metabolism, the responses of specific organs and cells should be considered when selecting a drug to block or reverse the metabolic reprogramming that occurs during fibrosis. Future research should aim to find a combination therapy or a precise targeted therapy that can successfully treat renal fibrosis, and then establish the best dosage, route of administration, and timing that provides the most benefit.

## Author Contributions

XZ contributed to the conception or design of the work, drafted, and revised articles. LJ provided guidance on setting up the article. ML helped modify the figure and table. XW helped to revised the article structure and partial language. YH drafted the manuscript. YD guided the idea of the article and edited, revised the manuscript and is responsible for all aspects of the article. All authors contributed to the article and approved the submitted version.

## Funding

This work was supported in part by grants from the Natural Science Foundation of Jilin Province (20190201248JC); the Jilin Province Development and Reform Commission (2018C052-9); and the Education Department of Jilin Province (JJKH20201050KJ).

## Conflict of Interest

The authors declare that the research was conducted in the absence of any commercial or financial relationships that could be construed as a potential conflict of interest.

## Publisher's Note

All claims expressed in this article are solely those of the authors and do not necessarily represent those of their affiliated organizations, or those of the publisher, the editors and the reviewers. Any product that may be evaluated in this article, or claim that may be made by its manufacturer, is not guaranteed or endorsed by the publisher.
